# An Essay on the Influence of Temperament in Modifying Dyspepsia or Indigestion

**Published:** 1831

**Authors:** 


AN ESSAY ON THE INFLUENCE OF TEMPERAMENT IN MODIFYING DYSPEPSIA OR INDIGESTION. By *Thomas Mayo*, M. D. &c. Octavo, pp. 144. London, 1831.

WE have here an addition to the numerous works on Indigestion with which the public has been inundated for the last ten or fifteen years. But it emanates from a respectable source, and is not a mere advertisement to catch practice, and thus gain two objects at once—money and experience—like a well-known surgeon, who announced a work on a particular operation which he had never performed, but which he hoped to have an opportunity of performing, through the instrumentality of said announcement.

Dr. Mayo, like most authors, deems it necessary to offer some reason for presenting his work to the world. The object of the Essay may be gathered from the following introductory passage.

"A patient labouring under indigestion applies to his medical adviser, or perhaps takes up one of the many valuable treatises by which the public has been familiarized with this interesting subject. He receives from his doctor, or he finds in his book, a series of rules, which seem to apply to many symptoms of his case. But he finds himself unrelieved, though he has employed remedies, directed against symptoms which have a real existence in himself, and which, he knows, his similarly disordered friend, Mr. A. or Mr. B., has found greatly beneficial. Or, reading many treatises and consulting many practitioners, he finds each of them assigning to his symptoms a different treatment.

Again, a young medical practitioner applies for instruction to some esteemed work on indigestion. He soon becomes aware, that it contains much valuable matter. But when he begins to practise from it, he finds the application of its rules involve him in numberless errors; and he begins to suspect that the work is valuable *only* as it sets him thinking for himself, or perhaps that it has no value at all. This surmise is greatly strengthened by his observing, that in many treatises, each evidently relating to the same class of symptoms, very different views of practice are entertained.

It is my object, as far as I can, to explain and to remove the above difficulties.—*Introd.* 2.

"*As far as I can*," was a most wise salvo in the foregoing passage—for, to remove the difficulties above stated, and reconcile the jarring practices of doctors or the theories of writers, is a task far beyond the powers of Dr. Mayo, or any other man.

After some observations, not very new, on the difference between medicine and the exact sciences, our author goes on to observe that, out of the mass of accumulated knowledge there do emerge some few well-ascertained principles which command general assent; while the great mass of materials lie unarranged and comparatively useless, the consequence of which is that many facts, after being ascertained and admitted, fade, and are lost to society. After some gentle censure on the merely practical man and the mere theorist, the author justly observes that, "one great object of every work should be to make the reader think for himself." We recommend the following passage to the perusal of those (and they form the majority of the profession) who despise study, and lean entirely on their own observations.

"The following principles of study are inculcated in the lectures of the late Dr. Armstrong, a practitioner, whose activity of mind fitted him in many respects to advance the science, and whose early death is a just subject of regret. I refer to them as accurately reported in the *Lancet*. After remarking contemptuously on Dr. Cullen, as being, what in the philosophical humility of his mind he was content to be, 'a man who introduced nothing original;' as if indeed to systematize the labours of others could involve no claim to intellectual merit, he states his own notions as to the extent and kind of information which a teacher should give, and which a medical student must be contented to receive. 'I wish you to understand,' says the Doctor, 'that these lectures will be neither elaborate nor learned in quotations, but will simply contain the results of my own observation and experience, laboriously conducted through a period of upwards of twenty years at the bed-side of sick individuals.'*

Now this is just the kind and extent of error which a little vanity, combined with a very dangerous misconception of the value of previously accumulated knowledge, is calculated to produce. It would almost appear, that there was a real advantage in knowledge of twenty years' standing over knowledge combining the results of these twenty years, with the collective experience of ages. But let the lecturer ask himself, how his modicum of practical information would ever have been obtained, but for these previously collected stores, which he affects to contemn, and which would probably assume a much more digested and valuable form, but for the mischievous sarcasms of similar reasoners. I know no other way to obviate this wayward tendency than that of giving just and reasonable views of the value of sound nosology as contrasted with precipitate theorizing. Had the ingenious writer just mentioned been aware how necessary it is for the practical utility of medical views, that they should rest on a broad basis of general principles obtained by comprehensive inquiry, he would, at the risk of being sometimes 'elaborate,' and even of indulging in quotations, have ventured upon a more extensive field than that supplied by his twenty years of personal experience. If Dr. Armstrong *himself* proceeded upon

---

"* Lancet, vols. v. vi. p. 361."

these principles through the whole of his own practice for those twenty years, what must we think of the perils of his patients during the first ten?" 10.

After some strictures on the danger of drawing descriptions of diseases from single cases, Dr. Mayo says that, "it would not be difficult to show that this oversight has vitiated the entire description of fever given by Dr. Armstrong." We knew Dr. Armstrong better than Dr. Mayo could possibly have done—and we are disposed to think that the late talented lecturer drew pictures of disease from a fertile imagination, or an exuberantly reproductive memory more frequently than from individual cases.

After these introductory observations, the author comes to the subject of the Essay. The first chapter is on the definition of indigestion, and its division relatively to *temperament.* He adopts, with the omission of a single word (primary) Dr. Paris's definition, and informs us that, with no disposition to depreciate the labours of other inquirers, he shall take up the subject of indigestion where they have left it. He thankfully refers to them for its general symptoms, while he endeavours to "show how the disease thus characterized may vary in different classes of men, according to certain combinations of qualities, physical, moral, and intellectual, called TEMPERAMENTS, by which these classes are distinguished." He adopts the ancient divisions, with the addition of the *nervous* temperament, and he then proceeds to consider indigestion in reference to bile, blood, phlegm, and nerve.

### Chap. II.—Description of Temperaments.

1. *Bilious Temperament.* This expression assumes the "tendency to a copious secretion of bile, in the person of whom it is predicated—and this is compatible with a good state of health. The morbid form of the temperament implies an obstructed, or vitiated, or excessive secretion."

"The bodily conformation of the bilious, is usually represented as rigid and spare, rather than full, or largely developed.

The effect of the bilious temperament upon complexion, is certainly to render it less clear, less brilliant, more sallow, than the same complexion is, whether dark or fair, under the sanguine temperament. In this sense alone we are authorized to describe it as influencing *colour.* The *texture* of the skin seems, on the other hand, very materially influenced by temperament. In the bilious, compared with the sanguine, it is harsh, and often arid. The sanguine temperament, as will be observed, gives a remarkable smoothness and elasticity, as well as brilliancy, to the skin.

'Cependant sans cette maudite bile, on ne gagne pas de grandes battailles,' said Buonaparte, according to M. Ségur, after complaining of its inconvenient effects in deranging his temper. Now, fanciful as this remark, and a great deal more of the same kind, may appear, I am not prepared to assert, that the universal impression as to the bilious predisposition possessing peculiar qualities of a moral and intellectual kind, is unfounded.

Among its most admitted traits, I should enumerate a gloomy but active imagination, a jealous, distrustful, and unsatisfied disposition, and an anxiously reflective cast of thought.

The dissatisfied nature of persons thus predisposed, would account for the stirring, restless, and ambitious course of action with which they are often charged. Such *would be* the prominent features of a life, in which it is supposed, that the *present* and *possessed* enjoyments become, *as such,* comparatively valueless.

One of the most distinctive features in this moral character, is the combination, which it often displays, of extreme and restless anxiety to be engaged in some employment, with a dull lethargic state of intellect, which entirely precludes useful exertion.

We may, indeed, generally observe, that the efforts of the bilious fall short of their aspirations.

But the works of MM. Richerand and Cabanis may be consulted, for a very diffuse and fanciful account of the temperament which I have here endeavoured to sketch. It has been *my* object to avoid conjectural remarks, and to limit myself to what appears extensively admitted or implied." 24.

### II.—NERVOUS TEMPERAMENT.

Our author acknowledges the difficulty of defining or describing this temperament. The principal characteristics of this, he thinks, may thus be stated.

"First, That it is highly susceptible of impressions. Secondly, That impressions once made are easily re-excited. Thirdly, That when it is in the state of morbid action proper to it, the solids of the body exhibit earlier and more marked phenomena than the fluids." 24.

Dr. Mayo illustrates the nervous temperament in the following manner. A person of vigorous intellect, and of a firm intrepid spirit, may be so organized that the loss of a trifling quantity of blood by venesection, shall produce syncope, even in a high state of health. Yet the same person (and a gallant officer is alluded to) may loose blood copiously by wounds in battle, without any such effect being produced.

"Now, in this case, two points may very fairly be assumed. First, That it is not the quantity of blood taken by the lancet, but some influence produced on the nervous system of the patient, that occasions the loss of power. Secondly, That the part of his nervous system, which first receives this impression, can scarcely be considered as having any reference to his intellectual or moral character. In this case, therefore, we must conclude, that the impression conveyed by the nerves is primarily of a physical kind; or, in other words, that it operates on a part of his system which does not concern him either as a moral or an intellectual agent. Whatever suspension of the powers of mind has ensued, is secondary. This first kind of nervousness I shall, therefore, call physical or bodily." 27.

The Doctor is a subtle casuist; and the above is a fair specimen of refined explanation. For our own parts, we would be tempted to solve the question in a much more concise, though perhaps not so physiological a manner. In battle, a man is in a state of excitement which will bear a loss of blood with little inconvenience, which, in a state of tranquillity, would cause faintness—in the same way that a man labouring under inflammatory fever would bear a depletion that would kill him almost, in a state of health. So, in great pain, opium may be taken to a far greater extent, without stupefaction, &c. than when no pain exists. Surely there need not be such an expenditure of words and ingenuity in stating these obvious facts founded on universal observation. We agree with our able author in the following remarks.

"Totally distinct from this is the susceptibility of nervous affection from moral impressions; a species of nervousness which I shall call moral. It is remarkably shewn in the bodily symptoms occasioned by timidity. But in each individual it must vary in form, according to the class of emotions, to which he is most predisposed.

Both of the foregoing species may be illustrated by the phenomena of hysteria. This affection may arise out of a series of moral feelings and impressions, influencing the nerves of the uterine system; which, once excited, occasion the various spasmodic symptoms distinguished by that name. Or it may arise from an irritation commencing in the womb, and not propagated from any prior emotion of the mind. In the first case, hysteria has a moral, in the second it has a physical origin. And how different will its treatment be in these two cases, if justly distinguished and appreciated!" 28.

It has been seen already, and will be still more evident in the sequel, that Dr. Mayo deprecates the idea or the practice of drawing general descriptions from individul cases. Yet one of the "three important heads" under which he discusses "nervous susceptibility" is founded on a single case.

"Thirdly, There are persons, in whom some operations of the intellect instantly produce a disturbance in the nervous system, which greatly interferes with the success of these operations. I have heard the sufferer describe this state of his nerves, as occasioning a cloud or mist suddenly to diffuse itself through his mind. In this way, again, nervous defects in articulation may be explained. A gentleman of very distinguished talent, singularly free from the two forms of nervousness above described, is well known by his friends to labour under the third. The occasions on which this defect has been observed in him, are usually those, in which the time for the performance of an intellectual task happens to be limited, the spectators or witnesses numerous, the object important. It is not that he feels more apprehensive of competition, or more anxious about the event, than other men similarly situated, but that his intellectual powers require to be humoured in every way, and are perplexed by circumstances, to which others of a more hardy, though less powerful intellect, would be impassive. Now this state of the understanding is liable, unless the above distinction be appreciated, to be treated as obstinacy, or as stupidity, or as deceit; because it wants those external phenomena of nervousness, which are palpable and conspicuous in the physical, and yet more so in the moral form.

The gentleman whose case I have adduced possesses the utmost steadiness of nerve, both physically and morally. His principles and his bodily constitution are alike sturdy and vigorous. It is only when his intellect has to energize, that the phenomena of nervous irritation are observable, and they are probably heightened by his complete consciousness of the powers which he possesses, and his occasional inability to exert them.

This, therefore, I shall call intellectual nervousness." 29.

### III.—Sanguine Temperament.

This temperament, which is discussed in little more than a page of very open type, is characterized by our author, as more nearly allied, in its ordinary form, to healthy, than to morbid actions. "It implies," says he, "a free and energetic circulation, a well-developed but firm muscular system, and a powerful conformation of the whole person. The complexion is usually florid; but the principal characteristic of the *temperament*, in *this* point, is brilliancy. The moral and intellectual properties of the sanguine are assumed to be such as correspond to a vigorous structure. These are vivacity, energy, and confidence."

### IV.—PHLEGMATIC OR SEROUS TEMPERAMENT.

We shall give this Section in the author's own words, lest it should be thought that we detracted from its merits by abbreviation in our own language.

" The main characteristic of this constitution is a deficiency of energy. It admits of a division into two heads : one embracing those cases in which the want of energy appears connected with a want of excitability ; the other comprising those in which it is connected with a want of power. The first of these divisions is well described by the popular term relaxed ; the other is the basis of the asthenic or feeble constitution, and, as such, is productive of the long and melancholy class of scrofulous affections. In both of these forms of the serous temperament the natural complexion is most frequently pallid. In both there is a remarkable absence of buoyancy and resiliency of habit. But the skin of the relaxed person, though pallid and bloodless, differs greatly from the unwholesome delicacy of the asthenic, and his muscular system is often even largely developed ; while, on the other hand, in the asthenic form, it usually happens, that the bodily structure, if not actually small, is rather fat than muscular. Persons of the relaxed habit are colourless ; while the complexion of the asthenic is often of a delicate redness. The pulses of both, except where fever occurs in the asthenic, are languid : but the languor of the relaxed seems connected with sluggishness, that of the asthenic with feebleness. In the relaxed person there may indeed be power ; but it is difficult to communicate an impulse which may bring that power into action. In the asthenic, the impulse communicated is felt indeed ; but it elicits no reaction, it merely exhausts.

In regard to moral and intellectual characteristics, as well as those which are purely physical, the serous temperament is well distinguished into the above varieties. The habitual sluggishness of simple relaxation, and, on the other hand, the feeble virtues and vices, and the languid conceptions of the asthenic class, are easily recognized. The character of Dr. Johnson was essentially of the first kind, which, it may be remembered, is compatible with *power*. Such were his strong but cumbrous exertions, slowly excited by the stimulus of necessity. Such, indeed, was the lazy application of extreme labour, which produced his dictionary. *He* well describes those painful exertions, under which the nobleman to whom he addressed himself, failed to assist him, until assistance was no longer wanted by him. Without some reference indeed to his languid temperament, it is difficult to account for the indifference with which a man so competent, and so disposed to investigate, could leave questions of extreme philological importance to pass unheeded, or to take their chance in the course of his execution of this extraordinary work.*

For instances of the asthenic class we must not explore the annals of intellectual or moral greatness. In its simple form, this kind of character passes through life unnoticed and unattractive. When complicated with the nervous temperament, it produces a kind of sensitive but powerless enthusiasm, which is in small repute as a quality of *men*,† but is often painfully beautiful in the other sex ; painfully, I say, because, in the latter case, we *can* give it our sympathy. For the want of defensive power which it implies, and which we are always

---

* " On this point Dr. Johnson is repeatedly censured by Horne Tooke.—The above remarks must be understood in reference to those operations of Dr. Johnson, in which even *he* was compelled to labour. The greater part of his works must have been *child's play* to his comprehensive understanding and richly furnished memory.

† " Such is the character of Wilfred in Rokeby."

disposed to treat contemptuously, when observed in man, is no blemish in the character of woman." 35.

The third chapter of the work embraces "a general sketch of indigestion," in which Dr. M. reminds the reader that "originality of matter is not his main object;" he therefore lays claim only to the utility of *applying* the labours of others. This chapter, in fact, is rather a critique on the works of Drs. Paris, Philip, and Johnson, than a delineation of the disease under consideration. It will, perhaps, on that very account, be more attractive to readers in general, who greedily devour opinions as to the merits of writers with whose productions they are familiar. The author shall speak freely for himself, even when we ourselves are somewhat depreciated.

"There is a remarkable diversity in the methods in which medical writers have respectively undertaken the history of indigestion. In the three able writers, whose works justly enjoy the highest present reputation on this subject, Dr. Paris, Dr. Wilson Philip, and Dr. Johnson, I find but little reference to temperament or constitution, as any ground of pathological distinctions. Dr. Wilson Philip furnishes a very masterly description of the disease. He has looked at his subject analytically, and he places his reader in full possession of *his* view of it. But this view is, in fact, just such a one as might be expected to occur to a clear medical eye, after a careful abstraction of those differences which a consideration of temperament would suggest. It will, I trust, appear in another part of this Essay, how necessary it is that such distinctions should be *entertained* and *admitted,* with a view to the complete development of Dr. Philip's subject. It is indeed curious, that he should not have applied such distinctions to indigestion, considering the avowed object of his treatise, 'to give arrangement to the affections termed nervous and bilious, and to ascertain the nature of the disease on which they depend.'

Without establishing any such division of the subject, Dr. Johnson's admirable work furnishes a much larger stock of materials for it, than that of Dr. Philip. The principal difference between the views of these two writers is, that Dr. Philip places before us a definite complaint,—Dr. Johnson describes a morbid habit. The first delineates an attack of dyspepsia, and follows this to its termination; the second draws from the life many characteristic features of a dyspeptic person.

Now it will be expedient to consider the ordinary form of the dyspeptic disease, as given by Philip, and the ordinary features of the dyspeptic patient, as pourtrayed by Dr. Johnson. This will form a useful basis for the more immediate subject of this Essay, the inquiry into those influences, by which temperament modifies the common phenomena of indigestion." 38.

A condensed sketch of Dr. Philip's views are then introduced without a single comment, after which Dr. Johnson comes in for some criticism as well as quotation. Dr. Paris has just right to complain, as not being noticed, either favourably or unfavourably, except as furnishing the best *nosological* character of dyspepsia.

"The indications of debility and irritability of the stomach and bowels, which Dr. Johnson considers the proximate cause of indigestion, are placed before us more loosely indeed, and with less attempt at system, but in a far more graphic way than by Dr. Philip. Dr. Johnson, indeed, enjoys the advantage, on which he frequently insists, of writing from his own feelings: an advantage, I beg leave to say, of a very questionable nature. In analysing disease, the physician has to deal with his own mind upon the same principles which he applies to the judgments formed by his patients and their sympathizing friends. He has *in them* to correct a tendency in constant operation to give unequal relative weight to the several features of the case, and he is sure to find that tendency active in regard to those points of disease with which their own personal experience may have most familiarized them. Now, the same difficulty besets *him* in keeping his own views steady and clear, when he has been himself a severe sufferer from any given illness, in regard to *that* illness; and his readers should always be on their guard, lest he should deal with his stores of knowledge with no greater discernment than a father may be observed to exhibit in regard to his children, when he has seen a great deal of some, and very little of others.

In common with most other writers on dyspepsia, Dr. Johnson directs his attention and his remarks chiefly to that form of the disease most frequent in the bilious habit. Though this, however, is his leading subject, he comprehends in many of his remarks, and, in his enumeration of the causes of morbid sensibility, a far wider field;* so that his work approaches more nearly than that of Dr. Philip to a view of indigestion classified according to the diversities of temperament.

Though no distinction founded expressly on such a theory is laid down by Dr. Johnson, he supplies some materials for it, so far as nervous indigestion may be contrasted with that of the bilious constitution. There is indeed much valuable practice suggested in relation to the nervous phenomena of the disease. Thus he animadverts on the system of Mr. Abernethy, where it well deserves animadversion, namely, as aiming at the recovery of health through a purgative and mercurial treatment alone. He similarly reprehends the abuse of Dr. Hamilton's purgative system; an abuse which may be entirely referred to the heedlessness of his followers, and not to any doctrines laid down in his invaluable work." 47.

We are inclined to think that Dr. Mayo has misapprehended Dr. Johnson, who, we may venture to assure him, does not claim any *advantage* from the circumstance of having suffered from dyspepsy. Whoever has felt the miseries of that disease, will never recal them to memory without horror—and will never boast of them as advantages. But Dr. Mayo seems to consider that a physician who has laboured under any particular disease, as for example, dyspepsia, is, in a measure, disqualified for writing on or treating the complaint—being in the predicament of a father evincing want of discernment to his own offspring. If Dr. Johnson has had but one patient all his life, and that one himself, there is little doubt but that his descriptions are merely auto-pictorial, and consequently are of no value beyond the individual from whom they are taken. But if, from personal sufferings, a man's attention be strongly directed to a subject, and that man has ample means of studying the said subject in others as well as in himself, we humbly conceive that the personal affliction is no disqualification. Was Dr. Bree's Treatise on Asthma (the best work in the English language on the subject) the worse because that able physician was a sufferer from the complaint?

Dr. Mayo proceeds to copy from Dr. Johnson's work four pages respecting the treatment of dyspepsy, concluding thus:—

"But Dr. Johnson's view of this subject would have been more comprehensive than it is, had it been constructed with a direct reference to a theory of

* "See particularly the valuable Remarks on the Phenomena of Repletion, and on those of Dyspepsia and Indigestion, from p. 18 to 43.

temperaments. Thus, if he had considered the large class in whose constitutions atony and relaxation predominate, and the liability of such persons, in an extreme degree, to the symptoms of dyspepsia, he surely would not have made this complaint identical with 'morbid sensibility of the stomach and bowels.'

In these persons it is morbid *insensibility* that constitutes the main feature of their ordinary indigestion. Such indeed is the usual state of the stomach induced by long continued gluttony; a vice unusual in highly sensitive constitutions. The dyspepsia of the glutton has no necessary connexion with any process of nervous irritation: it has duly ensued upon a long continued course of excessive stimulation, and consists in entire atony and languor." 51.

The foregoing passage convinces us that Dr. Mayo, however closely he may have studied *temperament*, has yet to learn the phenomena of dyspepsia —at least as far as regards the disease in gluttons and drunkards. Let any one of our readers, who has a patient of this description, pay attention for a single day to the symptoms, and if he do not see "*nervous irritation*," and nervous irritability as a prominent feature, we will acknowledge ourselves to be superficial observers. Debility and irritability have been recognized as parent and child, since the earliest records of medicine. Let Dr. Mayo exhibit some food of difficult digestion to one of his patients whose stomachs are atonic and languid from "excessive stimulation," and see the pain and the nervous commotion which will be excited in an organ which he maintains is in a state of "morbid *insensibility*." What would create no sensation in a healthy stomach, will here induce vomiting, or pain, or spasms, or a host of indescribable and horrible feelings in various parts of the body—yet this stomach, forsooth, is in a state of "morbid *insensibility!*"

Dr. Mayo next undertakes the defence of his friend Dr. Philip against the strictures of Dr. Johnson; and with this defence we find not the slightest fault. Valeat quantum valere debet.

In the 4th Chapter Dr. M. takes up the subject of "indigestion of the bilious temperament." This is the form of dyspepsy, Dr. M. observes, which is most commonly described by authors, because there is no other organ, he thinks, so influential in the production of the disease as the liver. The Doctor makes some sensible remarks on the connexion between dyspeptic ailments and mental emotions, but they are not particularly new, nor quite so well expressed as to be intelligible to all readers. This is a defect which pervades the work throughout. There is a very considerable obscurity in the language, though doubtless the ideas were clear enough in the author's own mind. The Doctor takes Dr. Philip's account of the disease and its treatment, making a few scanty remarks in the course of his quotations. Speaking of hypochondriasis, Dr. M. says he cannot agree with those who maintain that "*indigestion is by no means essential* to hypochondriasis." The Doctor then goes into a long disquisition to shew that, although there may be no symptom of indigestion present, yet we are not to rely on such evidence, as the patient is apt to deceive us as well as himself. All we shall say to this is—de non apparentibus et non existentibus eadem est ratio. We have seen many instances of hypochondriasis where there was not the slightest proof of indigestion, and where, on dissection, there was found organic diseases in the head or chest—none below the diaphragm.

" As melancholia* unfolds itself, the difference of treatment which given symptoms require, in relation to the accompanying temperament, becomes more important. The slow pulse, the extreme sense of lassitude, the moist relaxed skin, the entire absence of feverish excitation then observed in the bilious dyspeptic, symptoms which, it is to be observed, they possess in common with the phlegmatic, would incline us to the same active use of tonics and stomachics, as is really suitable to these symptoms, when met with in the latter temperament; and supposing we combine this practice with the occasional use of mild mercurials (a combination highly expedient under serous or phlegmatic indigestion) we shall find the bilious dyspeptic make accelerated progress towards the most dangerous stage of his disorder. For the mild mercurials excite, without giving it a due vent, the secretion of bile;—tonics lock up the enemy who requires to be expelled. The motions will become livid or dark; icteric symptoms will probably supervene; a suffusion will take place over the mind, as dark as the hue given to the face; and the mental affection, which overhangs the confirmed state of bilious dyspepsia, will become decided in character and very difficult of cure.

There is a change of symptoms often observable during the development of melancholia under bilious indigestion, which is very apt to give apparent reason for the use of strengthening and stimulating measures. In the case which I am supposing aperients *seem* rather prejudicial than salutary, from the extreme uneasiness which they produce: 'Omnibus fere melancholicis commune est,' says Lorry, 'ut statim ab alvo depositâ in pejus ruere sibi videantur.' Now the physician has to defend himself against the influence of this *appearance;* it does not, of itself, justify a change to the use of stimulants, though it may compel him to employ less irritating purgatives.

Some very able remarks are to be found in Dr. Johnson's work, in regard to change of scene and place, as it concerns the dyspeptic patient. And these may be applied, without much reserve, to the nervous and to the serous dyspepsia. But in regard to the bilious dyspeptic, when the worst (the mental) class of symptoms are impending, the most extreme caution is required in applying these remarks. Before the disorder has taken this direction, or again, when the melancholic symptoms are on the wane, at either end, as it were, of the disorder, travelling is very beneficial; but not in the intervening state. The serous, or nervous indigestion is better at almost any period for change of place, *as such;* for in these two latter kinds of disorder the stimulus thus applied often works a cure more promptly and effectually than any medical or dietetic expedient. But it is not thus with the *bilious dyspeptic;* 'cælum non animum mutat;' and in the meanwhile he postpones, or half performs those measures by which that part of his cure which depends upon medicine must be effected.

The 'bilious are usually incontinent:' and while travelling they are apt to avail themselves of the immediate benefit derived to their appetite from locomotion, and to take, with apparent impunity, articles of food, which they would otherwise be *unable*, and are still *unfit* to cope with.

The use of exercise is a most important consideration to the bilious temperament. In nervous indigestion it is often greatly overdone. Here it is seldom mischievous, except when applied at an unfit time in relation to meals; a point in determining which the idiosyncrasy of the patient must be consulted." 70.

We confess that we hardly comprehend Dr. Mayo's reasoning respecting the danger of travelling exercise in the *middle* of "bilious dyspepsy," while it is granted that the said exercise is advantageous "at *either end*, as

* " I know no other useful distinction between hypochondriasis and melancholia, than that of degree."

it were, of the disorder." There is no doubt that, in many cases of confirmed hypochondriasis, which is, indeed, but another name for insanity, "no earthly amusement, (as Dr. Johnson has acknowledged,) no change of scene, no mental impressions or excitement, no exercise of the body, can cheer the gloom that spreads itself over every object presented to the eye or the imagination! With them, change of place is only variety of woe!"* But, from some little experience and observation, we can assure Dr. Mayo and our readers in general, that travelling exercise, *judiciously conducted*, is free from danger in the beginning, middle, and end of hypochondriasis, as connected with "bilious dyspepsy." To the following observations, no one can object. They have been strongly urged by ourselves, on many occasions.

"In either case temperance, active exercise, and often a frequent repetition of mild aperients, are expedient, where the bilious temperament is strongly marked. Temperance, indeed, the rising from table with a stomach unoppressed, and a head not heated, is of paramount importance. And if the dark mental symptoms of this temperament are impending, such precautions are doubly requisite. For it is one of the afflicting circumstances of melancholia, that it deprives its victim of the moral energy by which alone he can persevere in measures requisite for his cure. In other diseases it is hard to find the appropriate remedy; in this, it is equally hard to apply the remedy when it has been found. And thus it happens, that we often see the unhappy sufferer unnerved in courage and enfeebled in intellect, revolving in a tedious cycle through a long series of medical advisers, without resolution enough to pay to any one of them consistent obedience.

With regard to the treatment of melancholia, viewed as an advanced stage of bilious indigestion, it must, from the outset of that treatment, be remembered, that the state of the patient has by that time become a very debilitated one.

The bilious temperament is not essentially a feeble one, but he, in whom the mental disease has supervened upon dyspepsia, has *become* asthenic. If his powers of *receiving food* are not greatly impaired, his powers of *obtaining nourishment* certainly are. Food, except when taken in the smallest quantities, generally oppresses him from the moment at which he has taken it, until some rapid aperient has freed him from it; and this state has, in most cases, continued long before the mind obtains attention as a seat of disease.

The risk of depletory measures, as tending to convert this secondary affection into an almost incurable state, the *démence* of the French writers, has accordingly become extreme. The lancet has no place here. The use of mercurials requires perseverance indeed, but caution and moderation. I have seen them, when pushed to salivation, change perversion of intellect into hopeless fatuity. This caution is the more required, in regard to our present subject, because melancholia or hypochondriasis, when a primary disease, and not the sequel or advanced stage of dyspepsia, bears on the whole more active depletion, than that acute and noisy form of insanity which belongs to the nervous temperament." 73.

From the fifth chapter on "Indigestion of the Nervous Temperament," we can only afford space for the following extract.

"A gentleman of a highly nervous temperament, placed in a situation of continued mental exertion, and much responsibility, in a West India island, was

* On Indigestion.

subjected, for some bilious symptoms, which were viewed without any reference to the predominant character of his constitution, to a severe mercurial treatment. He, at the same time, suffered from hemorrhoids, occasioning profuse discharges. His strength broken ; his circulation so disturbed that apoplexy at one moment, heart affection at another, seemed closely to impend; his skin constantly arid and giving no relief by perspiration to these last symptoms, he returned to this country. It is not my present purpose to detail the subsequent treatment of this case ; I wish to call my reader's attention to the fact, that it was found necessary, in the course of his treatment, to allow a far longer suspension of the action of the bowels than accorded with the general principles of practice, or than was comfortable to his own feelings, rather than expose him to the intense nervous excitement and exhaustion, which was occasioned by the process of fæcal evacuation, even when conducted in the mildest way. The relief, indeed, from feelings of obstruction, which purgatives were calculated to give him, his bowels being always in a confined state, was completely overborne by the attendant aggravation of *all* his other symptoms, such as flatulence, violent palpitation at the heart, with sense of approaching syncope, and vertiginous feelings in the head. All these sensations ensued upon the action of aperients so mild and so carefully chosen, as to imitate strictly the operations of nature, and yet to unload the bowels completely. Time, a patient endurance *on his part* of symptoms of which it was hazardous to attempt the complete relief, and a persevering abstinence *on the part of his physician* from such measures as might relieve present symptoms, and yet increase exhaustion; and, finally, a very cautious use of bark, ultimately restored him to health. The decisive and complete evidence of his recovery was, according to his own remark, the power of perspiring freely.

Now I do not adduce this case as an instance of dyspepsia, but as remarkably illustrating the effect of the nervous temperament in occasioning the ordinary functions of digestion, those indeed which we are usually compelled to excite and encourage in obviating morbid states of the digestive organs, to become, even in their moderate performance, a source of mischief through exhaustion.

This state, though strongly marked in regard to aperients, was not confined to *their* influence. Bark, as I have observed, promoted his recovery; but the difficulty of introducing bark without startling his nervous system, was extreme. I first decidedly succeeded in effecting this by means of inunction." 78.

We are obliged to pass over two chapters on indigestion as connected with the sanguineous and the phlegmatic temperament, as well as many ingenious observations on several other subjects, because our allotted space is already occupied. Dr. Mayo is evidently a well-educated and clever physician, and in drawing attention to TEMPERAMENT as influencing the phenomena and the treatment of diseases, he deserves the thanks of the profession. If we have differed from him on some points, we have only taken the same liberty which he has done, and which he had a right to do—and we hope in the same spirit of liberality and good feeling which characterizes his volume.

